# Robust PCA based method for discovering differentially expressed genes

**DOI:** 10.1186/1471-2105-14-S8-S3

**Published:** 2013-05-09

**Authors:** Jin-Xing Liu, Yu-Tian Wang, Chun-Hou Zheng, Wen Sha, Jian-Xun Mi, Yong Xu

**Affiliations:** 1 Bio-Computing Research Center, Shenzhen Graduate School, Harbin Institute of Technology, Shenzhen, China; 2 College of Information and Communication Technology, Qufu Normal University, Rizhao, China; 3 College of Electrical Engineering and Automation, Anhui University, Hefei, China; 4 Key Laboratory of Network Oriented Intelligent Computation, Shenzhen Graduate School, Harbin Institute of Technology, Shenzhen, China

## Abstract

How to identify a set of genes that are relevant to a key biological process is an important issue in current molecular biology. In this paper, we propose a novel method to discover differentially expressed genes based on robust principal component analysis (RPCA). In our method, we treat the differentially and non-differentially expressed genes as perturbation signals S and low-rank matrix A, respectively. Perturbation signals S can be recovered from the gene expression data by using RPCA. To discover the differentially expressed genes associated with special biological progresses or functions, the scheme is given as follows. Firstly, the matrix D of expression data is decomposed into two adding matrices A and S by using RPCA. Secondly, the differentially expressed genes are identified based on matrix S. Finally, the differentially expressed genes are evaluated by the tools based on Gene Ontology. A larger number of experiments on hypothetical and real gene expression data are also provided and the experimental results show that our method is efficient and effective.

## Background

One of the challenges in current molecular biology is how to find the genes associated with key cellular processes. Up to date, using microarray technology, these genes associated with a special biological process have been detected more comprehensively than ever before.

DNA microarray technology has enabled high-throughput genome-wide measurements of gene transcript levels [1,2], which is promising in providing insight into biological processes involved in gene regulation [3]. It allows researchers to measure the expression levels of thousands of genes simultaneously in a microarray experiment. Gene expression data usually contain thousands of genes (sometimes more than 10,000 genes), and yet only a small number of samples (usually less than 100 samples). Gene expression is believed to be regulated by a small number of factors (compared to the total number of genes), which act together to maintain the steady-state abundance of specific mRNAs. Some of these factors could represent the binding of one (or more) transcription factor(s) (TFs) to the promoter region(s) of the gene [4]. So, it can be assumed that the genes associated with a biological process are influenced only by a small subset of TFs [5]. Although the expression levels of thousands of genes are measured simultaneously, only a small number of genes are relevant to a special biological process. Therefore, it is important how to find a set of genes that are relevant to a biological process.

Various methods have been proposed for identifying differentially expressed genes from gene expression data. These methods can be roughly divided into two categories: univariate feature selection (UFS) and multivariate feature selection (MFS). The commonest scheme of UFS is utilized as follows. First, a score for each gene is independently calculated. Then the genes with high scores were selected [6]. The main virtues of UFS are simple, interpretable and fast. However, UFS has some drawbacks. For example, if each gene is independently selected from gene expression data, a large part of the mutual information contained in the data will be lost.

To overcome the drawbacks of UFS, the methods of MFS use all the features simultaneously to select the genes. So far, many mathematical methods for MFS, such as principal component analysis (PCA), independent component analysis (ICA), nonnegative matrix factorization (NMF), lasso logistic regression (LLR) and penalized matrix decomposition (PMD), have been devised to analyze gene expression data. For example, Lee *et al*. applied PCA to analyze gene expression data [7]. Liu *et al*. proposed a method of weighting principal components by singular values to select characteristic genes [8]. Probabilistic PCA was used to analyze gene expression data by Nyamundanda *et al*. [9]. Huang *et al*. used ICA to analyze gene expression data [10]. NMF was used to select the gene by Zheng *et al*. [11]. Liu *et al*. used LLR to select characteristic gene using gene expression data [12]. In [13], Witten *et al*. proposed penalized matrix decomposition (PMD), which was used to extract plant core genes by Liu *et al*. [14]. However, the brittleness of these methods with respect to grossly corrupted observations often puts its validity in jeopardy.

Recently, a new method for matrix recovery, namely robust PCA, has been introduced in the field of signal processing [15]. The problem of matrix recovery can be described as follows, assume that all the data points are stacked as column vectors of a matrix  D, and the matrix (approximately) have low rank:

(1) $$D=A_{0}+S_{0},$$

where $A_{0}$ has low-rank and $S_{0}$ is a small perturbation matrix. The robust PCA proposed by Candes *et al*. can recover a low-rank matrix $A_{0}$ from highly corrupted measurements  D[15]. Here, the entries in $S_{0}$ can have arbitrary large magnitude, and their support is assumed to be sparse but unknown.

Although the method has been successfully applied to model background from surveillance video and to remove shadows from face images [15], it's validity for gene expression data analysis is still need to be studied. The gene expression data all lie near some low-dimensional subspace [16], so it is natural to treat these genes data of non-differential expression as approximately low rank. As mentioned above, only a small number of genes are relevant to a biological process, so these genes with differential expression can be treated as sparse perturbation signals.

In this paper, based on robust PCA, a novel method is proposed for identifying differentially expressed genes. The differentially and non-differentially expressed genes are treated as perturbation signals  S and low-rank matrix  A. Firstly, the matrix  D of expression data is decomposed into two adding matrices  A and  S by using RPCA. Secondly, the differentially expressed genes are discovered according to the matrix  S. Finally, the differentially expressed genes are evaluated by the tools based on Gene Ontology. The main contributions of our work are as follows: firstly, it proposes, for the first time, the idea and method based on RPCA for discovery of differentially expressed genes; secondly, it provides a larger number of experiments of gene selection.

## Methods

### The definition of Robust PCA (RPCA)

This subsection simply introduces robust PCA (RPCA) proposed by Candes *et al*. [15]. Let ${||A||}_{*}:=\sum_{i}\sigma_{i}\left(A\right)$ denote the nuclear norm of the matrix  A, that is, the sum of its singular values, and let ${||S||}_{1}:=\sum_{ij}\left|S_{ij}\right|$ denote the $L_{1}$-norm of  S. Supposing that  D denotes the observation matrix given by Eq.(1), RPCA solves the following optimization problem:

(2) $$\begin{array}{cc} \text{minimize}\; & {||A||}_{*}+\lambda {||S||}_{1}\\ \text{subject to} & D=A+S \end{array},$$

where  λ is a positive regulation parameter. Due to the ability to exactly recover underlying low-rank structure in the data, even in the presence of large errors or outliers, this optimization is referred to as Robust Principal Component Analysis (RPCA).

For the RPCA problem Eq.(2), a Lagrange multiplier Y is introduced to remove the equality constraint. According to [17], the augmented Lagrange multiplier method on the Lagrangian function can be applied:

(3) $$L\left(A,S,Y,\mu \right)={||A||}_{*}+\lambda {||S||}_{1}+\langle Y,D-A-S\rangle +\frac{\mu }{2}{||D-A-S||}_{F}^{2},$$

where  μ is a positive scalar and ${||•||}_{F}^{2}$ denotes the Frobenius norm. Lin et al. gave a method for solving the RPCA problem, which is referred to as the inexact ALM (IALM) method [17]. The details of this algorithm can be seen in [17].

### The RPCA model of gene expression data

Considering the matrix  D of gene expression data with size m×n, each row of  D represents the transcriptional responses of a gene in all the  n samples, and each column of  D represents the expression levels of all the  m genes in one sample. Without loss of generality, m≫n, so it is a classical small-sample-size problem.

**Figure 1:**
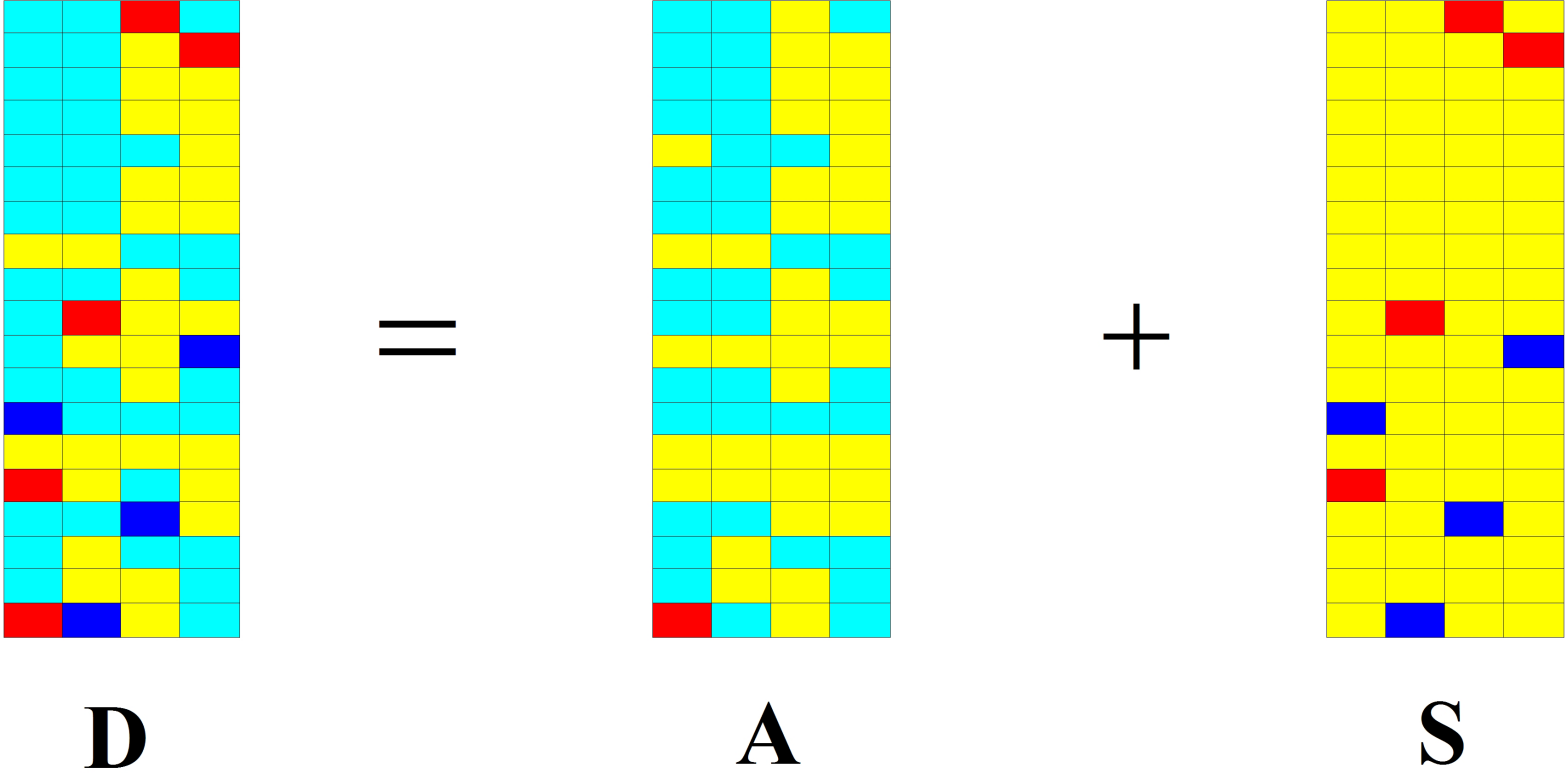
**The RPCA model of microarray data**. The white and yellow blocks denote zero and near-zero in this figure. Red and blue blocks denote the perturbation signals.

Our goal of using RPCA to model the microarray data is to identify these significant genes. As mentioned in Introduction, it is reasonable to view the significant genes as sparse signals, so the differential ones are viewed as the sparse perturbation signals  S and the non-differential ones as the low-rank matrix  A. Consequently, the genes of differential expression can be identified according to the perturbation signals  S. The RPCA model of microarray data is shown in Figure 1. The white and yellow blocks denote zero and near-zero in Figure 1. Red and blue blocks denote the perturbation signals. As shown in Figure 1, the matrix  S of differentially expressed genes (red or blue block) can be recovered from the matrix  D of gene expression data.

Suppose the matrix decomposition D=A+S has been done by using RPCA. By choosing the appropriate parameter  λ, the sparse perturbation matrix  S can be obtained, i.e., most of entries in  S are zero or near-zero (as white and yellow blocks shown in Figure 1). The genes corresponding to non-zero entries can be considered as ones of differential expression.

### Identification of differentially expressed genes

After observation matrix has been decomposed by using RPCA, sparse perturbation matrix  S can be obtained. Therefore the differentially expressed genes can be identified according to sparse matrix  S.

Denote the perturbation vector associated with  i-th sample as:

(4) $$S_{i}={\left[s_{1i},s_{2i},\cdots \;,s_{mi}\right]}^{T},\begin{array}{cc} & i=1,\cdots \;,n \end{array}.$$

Then the sparse matrix  S can be expressed as follows:

(5) $$S=\left[S_{1},\cdots \;,S_{n}\right].$$

So the sparse matrix  S can be denoted as:

(6) $$S=\left[\begin{array}{cccc} s_{11} & s_{12} & \cdots \; & s_{1n}\\ s_{21} & s_{22} & \cdots \; & s_{2n}\\ \vdots & \vdots & \ddots & \vdots \\ s_{m1} & s_{m2} & \cdots \; & s_{mn} \end{array}\right].$$

The differentially expressed genes can be classified into two categories: up-and down-regulated ones [18], which are reflected by the positive and negative entries in the sparse matrix  S. Here, to discover the differentially expressed genes, only the absolute value of entries in  S need to be considered. Then the following two steps are executed: firstly, the absolute values of entries in the sparse matrix  S are find out; secondly, to get the evaluating vector $\tilde{S}$, the matrix is summed by rows. Mathematically, it can be expressed as follows:

(7) $$\tilde{S}={\left[\begin{array}{ccc} \overset{n}{\underset{i=1}{\sum }}\left|s_{1i}\right| & \cdots \; & \overset{n}{\underset{i=1}{\sum }}\left|s_{mi}\right| \end{array}\right]}^{T}.$$

Consequently, to obtain the new evaluating vector  Ŝ, which is sorted in descending order. Without loss of generality, suppose that the first $c_{1}$ entries in  Ŝ are non-zero, that is,

(8) $$Ŝ={\left[\begin{array}{cc} \begin{array}{ccc} {ŝ}_{1}, & \cdots \;, & {ŝ}_{c_{1}}, \end{array} & \underset{m-c_{1}}{\underset{︸}{\begin{array}{ccc} 0, & \cdots \;, & 0 \end{array}}} \end{array}\right]}^{T}.$$

Generally, the larger the element in  Ŝ is, the more differential the gene is. So, the genes associated with only the first num ($num\leq c_{1}$) entries in  Ŝ are picked out as differentially expressed ones.

## Results and discussion

This section gives the experimental results. Firstly, in the first subsection, hypothetical data are exploited to clarify how to set the parameter λ. Secondly, in the second subsection, our method is compared with the following methods on the real gene expression data of plants responding to abiotic stresses: (a) PMD method using the left singular vectors $\left\{u_{k}\right\}$ to identify the differentially expressed genes (proposed by Witten *et al*. [13]); (b) SPCA method using all the PCs of SPCA (proposed by Journée *et al*. [19]) to identify the differentially expressed genes. Finally, in the third subsection, the three methods are compared on the real gene expression data of colon tumor.

### Experimental results on hypothetical data

Matrices randomly generated will be used for the simulation experiments. The true solution is denoted by the ordered pairs $\left(A^{*},S^{*}\right)$, which are generated by using the method in [17]. The rank-r matrix $A^{*}\in R^{m\times n}$ is generated as $A^{*}=LR^{T}$, where  L and  R are independent m×r and n×r matrices, respectively. Elements of  L and  R are i.i.d. Gaussian random variables with zero mean and unit variance. $S^{*}\in {\left\{-1,0,1\right\}}^{m\times n}$ is a sparse matrix whose support is chosen uniformly at random, and whose non-zero entries are i.i.d. uniformly in the space $R^{m\times n}$.  μ denotes the sparse degree of matrix $S^{*}$, which is defined as the number of non-zero entries divided by the number of all the entries. The matrix $D=A^{*}+S^{*}$ is the input data to the RPCA. To evaluate the identification performance of RPCA, $Acc_{S}$ denotes the recognition accuracy of matrix  S, which is defined as follows.

(9) $$Acc_{S}=\frac{Number\;of\;correct\;identified\;entries\;in\;S}{Number\;of\;entries\;in\;S},$$

where correct identified entries mean that the identified entries in  S approximately equal to the ones in $S^{*}$.

In [17,20], a fixed regulation parameter $\lambda ={\left(c*\text{max}\left(m,n\right)\right)}^{-1/2}$ is used, where c=1.0. In order to clarify how to set  λ, the following two different cases are considered: first, m=n; second, m>n, the small-size-sample problem.

### Results while m=n

In this experiment, let $m=n=\begin{array}{ccc} 500, & 1000 & \begin{array}{cc} or & 2000 \end{array} \end{array}$, $rankn=\begin{array}{ccc} 0.05 & or & 0.1 \end{array}$, $\begin{array}{cc} \begin{array}{cc} \mu =0.05 & or \end{array} & 0.1 \end{array}$. Table 1 lists the recognition results with different  c. As Table 1 listed, when c=0.2, the recognition accuracy $Acc_{S}$ can be achieved above 90%. When c≥0.3, the matrix  S can be completely identified, i.e. $Acc_{S}=\text{1}00\%$.

**Table 1:** The recognition accuracy $Acc_{S}$ with different  c

n	500				1000				2000			
rank/n	0.05	0.05	0.10	0.10	0.05	0.05	0.10	0.10	0.05	0.05	0.10	0.10
μ	0.05	0.10	0.05	0.10	0.05	0.10	0.05	0.10	0.05	0.10	0.05	0.10
c												

0.1	1.00	0.30	0.96	0.02	1.00	0.64	1.00	0.07	1.00	0.71	1.00	0.08
0.2	1.00	1.00	1.00	0.92	1.00	1.00	1.00	0.99	1.00	1.00	1.00	1.00
0.3	1.00	1.00	1.00	1.00	1.00	1.00	1.00	1.00	1.00	1.00	1.00	1.00
0.4	1.00	1.00	1.00	1.00	1.00	1.00	1.00	1.00	1.00	1.00	1.00	1.00
0.5	1.00	1.00	1.00	1.00	1.00	1.00	1.00	1.00	1.00	1.00	1.00	1.00
0.6	1.00	1.00	1.00	1.00	1.00	1.00	1.00	1.00	1.00	1.00	1.00	1.00
0.7	1.00	1.00	1.00	1.00	1.00	1.00	1.00	1.00	1.00	1.00	1.00	1.00
0.8	1.00	1.00	1.00	1.00	1.00	1.00	1.00	1.00	1.00	1.00	1.00	1.00
0.9	1.00	1.00	1.00	1.00	1.00	1.00	1.00	1.00	1.00	1.00	1.00	1.00
1.0	1.00	1.00	1.00	1.00	1.00	1.00	1.00	1.00	1.00	1.00	1.00	1.00

### Results while m>n

In this experiment, let m=10000,$rank=\begin{array}{ccc} 5 & or & 10 \end{array}$, $\begin{array}{cc} \begin{array}{cc} \mu =0.05 & or \end{array} & 0.1 \end{array}$ and  n increase from 10 to 100 with an interval 10. Table 2, 3, 4, 5 list the results. As tables 2 and 3 listed with rank=5, when n≥20, the recognition accuracy $Acc_{S}$ can be achieved above 90%. As tables 4 and 5 listed with rank=10, when n≥30, the recognition accuracy $Acc_{S}$ can be achieved above 90%. In words, to achieve the recognition accuracy $Acc_{S}$ above 90%,  n must be equal to or larger than three times of rank (n≥3*rank). As tables 2, 3, 4, 5 listed, by rows, the larger the number of column  n is, the higher the recognition accuracy $Acc_{S}$ can be achieved.

**Table 2:** The recognition accuracy $Acc_{S}$ with rank=5 and μ=0.05

c	n									
	
	10	20	30	40	50	60	70	80	90	100
0.1	1.00	0.30	0.96	0.02	1.00	0.64	1.00	0.07	1.00	0.71
0.2	1.00	1.00	1.00	0.92	1.00	1.00	1.00	0.99	1.00	1.00
0.3	1.00	1.00	1.00	1.00	1.00	1.00	1.00	1.00	1.00	1.00
0.4	1.00	1.00	1.00	1.00	1.00	1.00	1.00	1.00	1.00	1.00
0.5	1.00	1.00	1.00	1.00	1.00	1.00	1.00	1.00	1.00	1.00
0.6	1.00	1.00	1.00	1.00	1.00	1.00	1.00	1.00	1.00	1.00
0.7	1.00	1.00	1.00	1.00	1.00	1.00	1.00	1.00	1.00	1.00
0.8	1.00	1.00	1.00	1.00	1.00	1.00	1.00	1.00	1.00	1.00
0.9	1.00	1.00	1.00	1.00	1.00	1.00	1.00	1.00	1.00	1.00
1.0	1.00	1.00	1.00	1.00	1.00	1.00	1.00	1.00	1.00	1.00

**Table 3:** The recognition accuracy $Acc_{S}$ with rank=5 and μ=0.1

c	n									
	
	10	20	30	40	50	60	70	80	90	100
0.1	0.01	0.02	0.07	0.15	0.24	0.36	0.43	0.51	0.59	0.66
0.2	0.24	0.84	0.99	1.00	1.00	1.00	1.00	1.00	1.00	1.00
0.3	0.50	0.95	1.00	1.00	1.00	1.00	1.00	1.00	1.00	1.00
0.4	0.61	0.97	1.00	1.00	1.00	1.00	1.00	1.00	1.00	1.00
0.5	0.62	0.96	1.00	1.00	1.00	1.00	1.00	1.00	1.00	1.00
0.6	0.64	0.94	0.99	1.00	1.00	1.00	1.00	1.00	1.00	1.00
0.7	0.64	0.93	0.99	1.00	1.00	1.00	1.00	1.00	1.00	1.00
0.8	0.65	0.91	0.99	1.00	1.00	1.00	1.00	1.00	1.00	1.00
0.9	0.66	0.89	0.98	1.00	1.00	1.00	1.00	1.00	1.00	1.00
1.0	0.67	0.86	0.97	0.99	1.00	1.00	1.00	1.00	1.00	1.00

**Table 4:** The recognition accuracy $Acc_{S}$ with rank=10 and μ=0.05

c	n									
	
	10	20	30	40	50	60	70	80	90	100
0.1	0.00	0.06	0.50	0.92	0.99	1.00	1.00	1.00	1.00	1.00
0.2	0.06	0.61	0.99	1.00	1.00	1.00	1.00	1.00	1.00	1.00
0.3	0.15	0.77	0.99	1.00	1.00	1.00	1.00	1.00	1.00	1.00
0.4	0.27	0.74	0.98	1.00	1.00	1.00	1.00	1.00	1.00	1.00
0.5	0.40	0.67	0.96	1.00	1.00	1.00	1.00	1.00	1.00	1.00
0.6	0.50	0.63	0.93	0.99	1.00	1.00	1.00	1.00	1.00	1.00
0.7	0.59	0.60	0.88	0.98	1.00	1.00	1.00	1.00	1.00	1.00
0.8	0.66	0.59	0.82	0.97	1.00	1.00	1.00	1.00	1.00	1.00
0.9	0.71	0.61	0.76	0.94	0.99	1.00	1.00	1.00	1.00	1.00
1.0	0.75	0.65	0.72	0.90	0.98	1.00	1.00	1.00	1.00	1.00

**Table 5:** The recognition accuracy $Acc_{S}$ with rank=10 and μ=0.1

c	n									
	
	10	20	30	40	50	60	70	80	90	100
0.1	0.01	0.01	0.00	0.01	0.01	0.01	0.02	0.04	0.07	0.09
0.2	0.22	0.16	0.50	0.89	0.99	1.00	1.00	1.00	1.00	1.00
0.3	0.51	0.43	0.89	0.99	1.00	1.00	1.00	1.00	1.00	1.00
0.4	0.62	0.56	0.93	0.99	1.00	1.00	1.00	1.00	1.00	1.00
0.5	0.64	0.59	0.92	0.99	1.00	1.00	1.00	1.00	1.00	1.00
0.6	0.64	0.58	0.88	0.98	1.00	1.00	1.00	1.00	1.00	1.00
0.7	0.65	0.58	0.83	0.96	0.99	1.00	1.00	1.00	1.00	1.00
0.8	0.65	0.59	0.79	0.94	0.99	1.00	1.00	1.00	1.00	1.00
0.9	0.67	0.61	0.73	0.91	0.98	1.00	1.00	1.00	1.00	1.00
1.0	0.68	0.65	0.70	0.86	0.96	0.99	1.00	1.00	1.00	1.00

**Figure 2:**
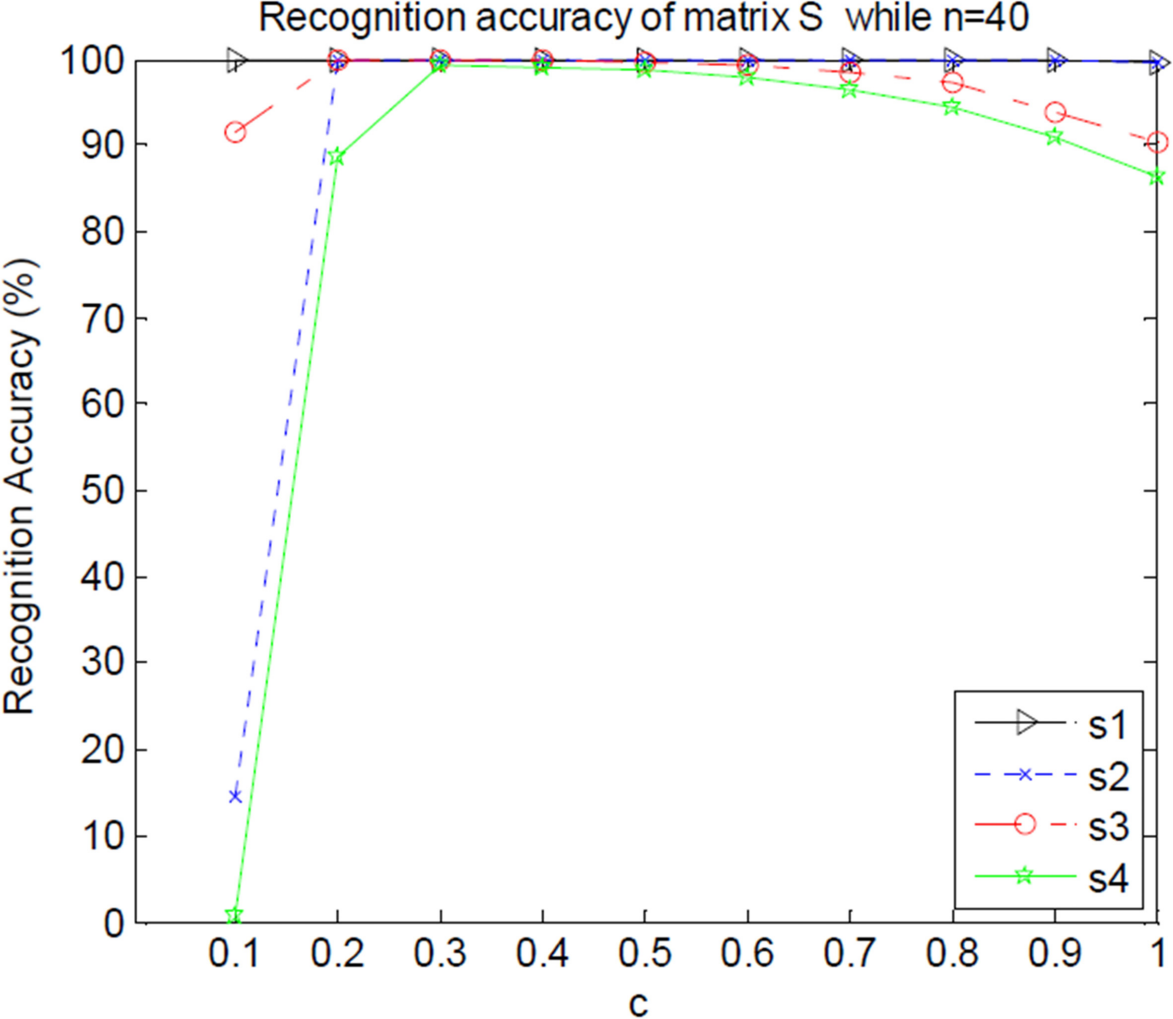
**The recognition accuracy of matrix  S with different  c**. s1 denotes the recognition accuracy series with rank=5 and μ=0.05. s2 denotes the recognition accuracy series with rank=5 and μ=0.1. s3 denotes the recognition accuracy series with rank=10 and μ=0.05. s4 denotes the recognition accuracy series with rank=10 and μ=0.1.

Now, we investigate how different  c influences the recovery accuracy $Acc_{S}$. For example, when n=40, Figure 2 shows the recognition accuracy $Acc_{S}$ with different  c. As shown in Figure 2, when c=0.3, the recognition of matrix  S can reach highest accuracy. With  c increasing, the recovery accuracy $Acc_{S}$ drops. For example, when c=1.0, s3 and s4 are degraded to 90%.

From these experiments, a conclusion can be drawn that when the optimal empirical value of  λ is given as: $\lambda ={\left(0.3*\text{max}\left(m,n\right)\right)}^{-1/2)}$, where the size of data matrix  D is m×n, the highest identification accuracy $Acc_{S}$ can be obtained.

### Experimental results on gene expression data of plants responding to abiotic stresses

Along with other two state-of-the-art methods, namely PMD and SPCA, used as comparison, three methods, including RPCA, are used to discover the differentially expressed genes responding to abiotic stresses based on real gene expression data.

### Data source

The raw data were downloaded from NASCArrays [http://affy.arabidopsis.info/] [21], which include two classes: roots and shoots in each stress. The reference numbers are: control, NASCArrays-137; cold stress, NASCArrays-138; osmotic stress, NASCArrays-139; salt stress, NASCArrays-140; drought stress, NASCArrays-141; UV-B light stress, NASCArrays-144; heat stress, NASCArrays-146. Table 6 lists the sample number of each stress type. There are 22810 genes in each sample. The data are adjusted for background of optical noise using the GC-RMA software by Wu *et al*. [22] and normalized using quartile normalization. The results of GC-RMA are gathered in a matrix for further processed.

**Table 6:** The sample number of each stress type in the raw data

Stress Type	cold	drought	salt	UV-B	heat	osmotic	control
Number of Samples	6	7	6	7	8	6	8

### Selection of the parameters

In this paper, for PMD method, the $L_{1}$-norm of u is taken as the penalty function, i.e. ${||u||}_{1}\leq \alpha_{1}$. Because of $1\leq \alpha_{1}\leq \sqrt{m}$, let $\alpha_{1}=\alpha *\sqrt{m}$, where $1\sqrt{m}\leq \alpha \leq 1$. For simplicity, let p=1, that is, only one factor is used. The results with $L_{1}$-norm (${||z||}_{1}=\sum_{i}\left|z_{i}\right|$) and $L_{0}$-norm (${||z||}_{0}$, i.e. the number of nonzero coefficients, or cardinality) penalty in SPCA are similar, which is also shown in [19], so $L_{0}$-norm penalty and the parameter  γ are taken in SPCA. For a fair comparison, 500 genes are roughly selected by these methods via choosing appropriate parameters  α and  γ of the two methods, PMD and SPCA, which are listed in Table 7 for different data set. As the first subsection of experiments mentioned, while c=0.3, RPCA gives the optimization results. Then, according to methods section, the first 500 genes are selected.

**Table 7:** The values of  α and  γ on different data set

*Stress*	*shoot*	*shoot*	*root*	*root*
	
	PMD	SPCA	PMD	SPCA
	α	γ	α	γ
drought	0.0928	0.4224	0.0999	0.4065
salt	0.0924	0.4920	0.1057	0.5261
UV-B	0.1036	0.4505	0.0966	0.4329
cold	0.1026	0.4660	0.0983	0.4726
heat	0.0765	0.3770	0.0931	0.3710
osmotic	0.1049	0.5139	0.0946	0.5338

### Gene ontology (GO) analysis

Recently, many tools have been developed for the functional analysis of large lists of genes [23,24]. Most of them focus on the evaluation of Gene Ontology (GO) annotations. GOTermFinder is a web-based tool that finds the significant GO terms shared among a list of genes, helping us discover what these genes may have in common. The analysis of GOTermFinder provides significant information for the biological interpretation of high-throughput experiments.

In this subsection, the genes identified by these methods, RPCA, PMD and SPCA, are sent to GOTermFinder [24], which is publicly available at http://go.princeton.edu/cgi-bin/GOTermFinder. Its threshold parameters are set as following: minimum number of gene products = 2 and maximum P-value = 0.01. Here, the key results are shown. Table 8 lists the terms of Response to abiotic stimulus (GO:0009628), whose background frequency in TAIR set is 1539/29556 (5.2%). Response to abiotic stimulus is the ancestor term of all the abiotic stresses. In GOTermFinder, a p-value is calculated using the hyper-geometric distribution, its details can be seen in [24]. Sample frequency denotes the number of genes hit in the selected genes, such as 107/500 denotes 107 genes associated with the GO term in 500 ones selected by these methods. As listed in Table 8, all the three experimented methods, PMD, SPCA and RPCA, can extract the significant genes with very lower P-value, as well as very higher sample frequency. In Table 8, the superior results are in bold type. In the twelve items, there is only one of them (cold on root) that PMD is equal to our method. In other items, our method is superior to SPCA and PMD.

**Table 8:** Response to abiotic stimulus (GO:0009628)

*Stress* *type*		*PMD*		*SPCA*		*RPCA*	
		
		P-value	Sample frequency	P-value	Sample frequency	P-value	Sample frequency
drought	s	3.91E-34	107/500 (21.4%)	7.5E-21	87/500 (17.4%)	1.09E-45	**122/500 (24.4%)**
drought	r	1.78E-10	68/500 (13.6%)	4.14E-08	63/500 (12.6%)	1.03E-27	**98/500 (19.6%)**
salt	s	9.93E-39	113/500 (22.6%)	9.83E-33	105/500 (21.0%)	1.35E-55	**134/500 (26.8%)**
salt	r	1.36E-15	78/500 (15.6%)	6.18E-12	71/500 (14.2%)	1.65E-22	**90/500 (18.0%)**
UV-B	s	1.76E-13	74/500 (14.8%)	7.84E-23	90/500 (18.0%)	5.9E-41	**116/500 (23.2%)**
UV-B	r	5.3E-10	67/500 (13.4%)	8.00 E-4	52/500 (10.4%)	4.73E-29	**100/500 (20.0%)**
cold	s	5.82E-35	106/500 (21.6%)	1.17E-19	85/500 (17.0%)	2.13E-46	**123/500 (24.6%)**
cold	r	2.74E-23	**91/500 (18.2%)**	4.1E-19	84/500 (16.8%)	4.02E-23	**91/500 (18.2%)**
heat	s	1.44E-24	93/500 (18.6%)	4.64E-22	89/500 (17.8%)	7.46E-55	**133/500 (26.6%)**
heat	r	1.41E-15	78/500 (15.6%)	1.35E-08	64/500 (12.8%)	1.07E-34	**108/500 (21.6%)**
osmotic	s	6.55E-38	112/500 (22.4%)	2.02E-18	83/500 (16.6%)	6.83E-54	**132/500 (26.4%)**
osmotic	r	1.4E-14	76/500 (15.2%)	2.87E-17	81/500 (16.2%)	9.98E-35	**108/500 (21.6%)**

In this table, 's' denotes the shoot samples; 'r' denotes the root samples.

**Figure 3:**
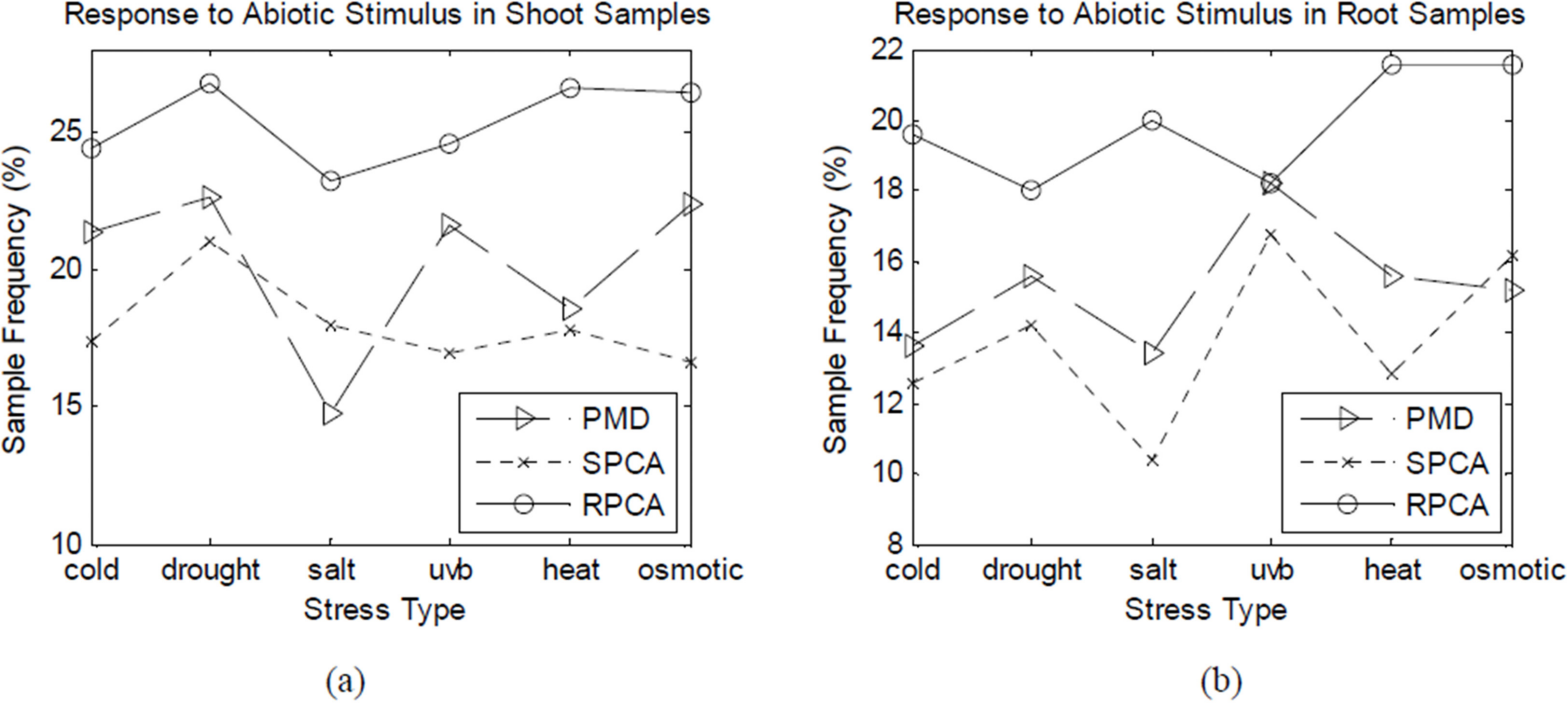
**The sample frequency of response to abiotic stimulus**.

Figure 3 shows the sample frequency of response to abiotic stimulus (GO:0009628) given by the three methods. From Figure 3(a), RPCA method outperforms others in all the data sets of shoot samples with six different stresses. Figure 3(b) shows that only in cold-stress data set of root samples, PMD is equal to our method and they are superior to SPCA. In other data sets, our method is superior to the others.

The characteristic terms are listed in Table 9, in which the superior results are in bold type. As listed in Table 9, PMD method outperforms SPCA and our method in three items, such as drought in shoot, salt in root and cold in root, among the whole items. However, it shows that, on one of the twelve items (osmotic in shoot), our method has the same competitive result as PMD, while both methods are superior to SPCA. In other eight items, our method excels PMD and SPCA methods. In addition, on all the characteristic items, our method has superiority over SPCA.

**Table 9:** Characteristic terms selected from GO by algorithms

Stress type		GO Terms	Background frequency	Sample frequency		
				
				***PMD***	***SPCA***	***RPCA***
drought	s	GO:0009414 response to water deprivation	207/29887 (0.7%)	**47/500 (9.4%)**	23/500 (4.6%)	34/500 (6.8%)
drought	r	GO:0009415 response to water deprivation	207/29887 (0.7%)	26/500 (5.2%)	24/500 (4.8%)	**30/500 (6.0%)**
salt	s	GO:0009651 response to salt stress	395/29887 (1.3%)	41/500 (8.2%)	28/500 (5.6%)	**48/500 (9.8%)**
salt	r	GO:0009651 response to salt stress	395/29887 (1.3%)	**33/500 (6.6%)**	22/500 (4.4%)	31/500 (6.2%)
UV-B	s	GO:0009416Response to light stimulus	557/29887 (1.9%)	23/500 (4.6%)	30/500 (6.0%)	**42/500 (8.4%)**
UV-B	r	GO:0009416Response to light stimulus	557/29887 (1.9%)	24/500 (4.8%)	none	**36/500 (7.2%)**
cold	s	GO:0009409 response to cold	276/29887 (0.9%)	44/500 (8.8%)	34/500 (6.8%)	**58/500 (11.6%)**
cold	r	GO:0009410 response to cold	276/29887 (0.9%)	**43/500 (8.6%)**	33/500 (6.6%)	38/500 (7.6%)
heat	s	GO:0009408 response to heat	140/29887 (0.5%)	45/500 (9.0%)	30/500 (6.0%)	**47/500 (9.4%)**
heat	r	GO:0009409 response to heat	140/29887 (0.5%)	43/500 (8.6%)	28/500 (5.6%)	**48/500 (9.6%)**
osmotic	s	GO:0006970 response to osmotic stress	474/29887 (1.6%)	**55/500 (11.0%)**	29/500 (5.8%)	**55/500 (11.0%)**
osmotic	r	GO:0006970 response to osmotic stress	474/29887 (1.6%)	39/500 (7.8%)	27/500 (5.4%)	**41/500 (8.2%)**

In this table, 's' denotes the shoot samples; 'r' denotes the root samples; 'none' denotes that the algorithm cannot give the GO terms.

From the results of experiments, it can be concluded that our method is efficient and effective.

### Experimental results on colon data

The three methods, SPCA, PMD and RPCA, are compared on colon cancer data set [25]. Colon cancer is the fourth most common cancer for males and females and the second most frequent cause of death.

### Data source

The raw data were downloaded from http://genomics-pubs.princeton.edu/oncology/affydata/I2000.html, which include gene expression levels for 2000 gene and contain 40 tumor and 22 normal tissue samples.

### Selection of the parameters

In this subsection, for PMD method, the $L_{1}$-norm of u is taken as the penalty function, i.e. ${||u||}_{1}\leq \alpha_{1}$. Let $\alpha_{1}=\alpha *\sqrt{m}$, where $1\sqrt{m}\leq \alpha \leq 1$. For SPCA method, let p=1, that is, only one factor is used. $L_{0}$-norm penalty and the parameter  γ are taken in SPCA. For a fair comparison, 100 genes are roughly selected by these methods via choosing appropriate parameters. PMD and SPCA use the parameters α=0.2351 and γ=0.4306 on colon data set, respectively. As the first subsection of experiments mentioned, while c=0.3, RPCA gives the optimization results. Then, according to Methods section,the first 100 genes are selected using our method.

### Gene ontology (GO) analysis

The genes identified by these methods, RPCA, PMD and SPCA, are evaluated by using AmiGO [26]. Its threshold parameters are set as following: minimum number of gene products = 2 and maximum P-value = 0.1. A number of lines of evidence suggest that immune, stimulus and tumor have affinity, so Table 10 lists the key results: the terms of Response to stimulus (GO:0050896) and Immune system process (GO:0002376). As listed in Table 10, RPCA outperforms its competitive methods with higher sample frequency.

**Table 10:** Characteristic terms selected from GO on colon data

*GO Term*	*Response to stimulus*	*Immune system process*
Accession No.	GO:0050896	GO:0002376
Background frequency	32294/155706 (20.7%)	7011/155706 (4.5%)
P-value(RPCA)	1.76E-10	5.74E-09
Sample frequency (RPCA)	38/57 (66.7%)	19/57 (33.3%)
P-value(SPCA)	8.71E-06	2.95E-04
Sample frequency (SPCA)	32/57 (56.1%)	14/57 (24.6%)
P-value(PMD)	7.93E-04	8.27E-01
Sample frequency (PMD)	27/51 (52.9%)	9/51 (17.6%)

### Function analysis

Table 11 lists the top 30 genes selected by using RPCA. To further study the biology functions of the selected genes, we also make the network analysis of the top 100 genes selected by our algorithm using the GeneMANIA tool [27] on the Web sitehttp://genemania.org/. The result is listed in Table 12. From the table it can be seen that there are 215 genes of this chip participating in the cytokine-mediated signalling pathway, in which there are 21 genes discovered by our method. This pathway has the lowest p-value. It is considered as the most probable pathway with these top 100 genes. Recent findings also indicate that cytokine receptors can regulate immune cell functions by transcription-independent mechanisms [28]. Some other pathways with the most significance are also listed in Table 12.

**Table 11:** The top 30 genes of colon data selected by RPCA

*Gene No*.	*Sequence*	*Gene Name*
M27190	gene	Homo sapiens secretary pancreatic stone protein (PSP-S) mRNA, complete cds.
R89823	3' UTR	INORGANIC PYROPHOSPHATASE (Bos taurus)
M87789	gene	IG GAMMA-1 CHAIN C REGION (HUMAN).
T48904	3' UTR	HEAT SHOCK 27 KD PROTEIN (HUMAN).
M26383	gene	Human monocyte-derived neutrophil-activating protein (MONAP) mRNA, complete cds.
J00231	gene	Human Ig gamma3 heavy chain disease OMM protein mRNA.
X02761	gene	Human mRNA for fibronectin (FN precursor).
R80612	3' UTR	PHOSPHOLIPASE A2, MEMBRANE ASSOCIATED PRECURSOR (HUMAN).
M31994	gene	Human cytosolic aldehyde dehydrogenase (ALDH1) gene, exon 13.
T47377	3' UTR	S-100P PROTEIN (HUMAN).
X02492	gene	INTERFERON-INDUCED PROTEIN 6-16 PRECURSOR (HUMAN); contains L1 repetitive element.
M94132	gene	Human mucin 2 (MUC2) mRNA sequence.
X67325	gene	H.sapiens p27 mRNA.
D28137	gene	Human mRNA for BST-2, complete cds.
L05144	gene	PHOSPHOENOLPYRUVATE CARBOXYKINASE, CYTOSOLIC (HUMAN); contains Alu repetitive element; contains element PTR5 repetitive element.
X02874	gene	Human mRNA for (2'-5') oligo A synthetase E (1,6 kb RNA).
T55117	3' UTR	ALPHA-1-ANTITRYPSIN PRECURSOR (HUMAN).
M19045	gene	Human lysozyme mRNA, complete cds.
Y00711	gene	L-LACTATE DEHYDROGENASE H CHAIN (HUMAN);.
X60489	gene	Human mRNA for elongation factor-1-beta.
T57780	3' UTR	IG LAMBDA CHAIN C REGIONS (HUMAN).
T60778	3' UTR	MATRIX GLA-PROTEIN PRECURSOR (Rattus norvegicus).
H58397	3' UTR	TRANS-1, 2-DIHYDROBENZENE-1, 2-DIOL DEHYDROGENASE (HUMAN).
L08044	gene	Human intestinal trefoil factor mRNA, complete cds.
M18216	gene	Human nonspecific cross reacting antigen mRNA, complete cds.
K03474	gene	Human Mullerian inhibiting substance gene, complete cds.
L33930	gene	Homo sapiens CD24 signal transducer mRNA, complete cds and 3' region.
T48014	3' UTR	HEMOGLOBIN ALPHA CHAIN (HUMAN).
H73908	3' UTR	METALLOTHIONEIN-IA (Bos taurus)
R70030	3' UTR	IG MU CHAIN C REGION (HUMAN).

**Table 12:** Pathway analysis of the top 100 genes selected by RPCA on colon data

rank	Go annotation	Q-value	Genes in network	Genes in genome
1	cytokine-mediated signalling pathway	2.27E-20	21	215
2	cellular response to cytokine stimulus	1.70E-19	21	244
3	response to cytokine stimulus	2.62E-18	21	283
4	type I interferon-mediated signalling pathway	1.61E-17	14	71
5	cellular response to type I interferon	1.61E-17	14	71
6	response to type I interferon	1.67E-17	14	72
7	interferon-gamma-mediated signalling pathway	2.60E-08	9	77
8	cellular response to interferon-gamma	3.64E-08	9	81
9	response to interferon-gamma	1.04E-07	9	92
10	response to other organism	3.69E-05	10	243

## Conclusion

In this paper, a novel RPCA-based method of discovering differentially expressed genes was proposed. It combined RPCA and sparsity of gene differential expression to provide an efficient and effective approach for gene identification. Our method mainly included the following two steps: firstly, the matrix  S of differential expression was discovered from gene expression data matrix by using robust PCA; secondly, the differentially expressed genes were discovered according to matrix  S. The experimental results on real gene data showed that our method outperformed the other state-of-the-art methods. In future, we will focus on the biological meaning of the differentially expressed genes.

## Competing interests

The authors declare that they have no competing interests.

## Declarations

The publication costs for this article were funded by fund for China Postdoctoral Science Foundation Funded Project, No. 2012M510091.

This article has been published as part of *BMC Bioinformatics *Volume 14 Supplement 8, 2013: Proceedings of the 2012 International Conference on Intelligent Computing (ICIC 2012). The full contents of the supplement are available online at http://www.biomedcentral.com/bmcbioinformatics/supplements/14/S8.
